# Efficacy of Tocilizumab in Refractory Graves Orbitopathy From Real-World Clinical Practice: An Observational Study

**DOI:** 10.1210/jendso/bvae193

**Published:** 2024-11-02

**Authors:** Mingyang Wang, Bixuan Qin, Cuihong Liu, Honglei Liu, Dongmei Li

**Affiliations:** Beijing Tongren Eye Center, Beijing Tongren Hospital, Capital Medical University, Beijing 100730, China; Shaanxi Eye Hospital, Xi’an People’s Hospital (Xi’an Fourth Hospital), Affiliated People's Hospital of Northwest University, Xi’an 710199, China; Shaanxi Eye Hospital, Xi’an People’s Hospital (Xi’an Fourth Hospital), Affiliated People's Hospital of Northwest University, Xi’an 710199, China; Shaanxi Eye Hospital, Xi’an People’s Hospital (Xi’an Fourth Hospital), Affiliated People's Hospital of Northwest University, Xi’an 710199, China; Beijing Tongren Eye Center, Beijing Tongren Hospital, Capital Medical University, Beijing 100730, China; Department of Ophthalmology, Aier Eye Hospital Group Co., Ltd. Beijing Aier Eye Hospital, Beijing 100101, China; Department of Ophthalmology, Jinan University, Guangzhou, Guangdong 510071, China

**Keywords:** Graves orbitopathy (GO), tocilizumab (IL-6 inhibitor, TCZ), thyrotropin receptor antibody, clinical activity score, exophthalmos, visual acuity

## Abstract

**Context:**

The efficacy of tocilizumab (TCZ) in treating Graves orbitopathy (GO) remains uncertain due to the small sample sizes of earlier studies, and there is a lack of research on the drug for juvenile GO.

**Objective:**

To evaluate the effectiveness of TCZ in treating GO that is resistant to conventional therapy.

**Design:**

This observational study at a tertiary care center included 79 Chinese GO patients, 15 of whom were pediatric patients, with 52 of these patients having moderate to severe active GO (all adult patients having steroid-resistant GO). Intravenous infusion of TCZ 8 mg/kg was given every 28 days for 4 months. Changes from baseline in visual acuity (VA), intraocular pressure (IOP), proptosis, clinical activity score (CAS), and thyrotropin receptor antibody (TRAb) levels throughout TCZ therapy were assessed at baseline (T0), the fifth month (T4), and follow-up (T5). Additionally, improvements in CAS by at least 2 points and CAS < 4 points at T4 and T5 were evaluated.

**Results:**

Significant improvements were found in VA, IOP, proptosis, CAS, and TRAb levels in the adult group, and proptosis in the pediatric group at T5 (*P* < .05). Additionally, significant improvements were identified in TRAb levels and CAS (active GO at T0) in the pediatric group at T4 (*P* < .05). In the adult and pediatric group with active GO at T5, 71.4% and 60% experienced a decrease in CAS by ≥ 2 points, respectively; 89.3% and 60% achieved the response criterion of low activity disease (CAS < 4 points), respectively.

**Conclusion:**

TCZ emerged as a valuable therapeutic option for Chinese patients with active, corticosteroid-resistant, moderate to severe GO.

Graves orbitopathy (GO) is an autoimmune inflammatory disease that affects the orbital fat and muscles, and it is diagnosed in 30% to 50% of patients with Graves disease (GD) [1, 2]. This disease can manifest in a variety of forms, ranging from inflammation of the extraocular muscles to severe cases of constant diplopia, protrusion, sight threatening with dysthyroid optic neuropathy (DON), and corneal breakdown. Approximately 5% of patients with GD are affected by moderate to severe orbitopathy [1].

In cases of moderate to severe or vision-threatening active GO, it is crucial to systematically control the patient's thyroid hormones to prevent exacerbations or relapses. High-dose corticosteroids remain the gold standard of treatment, accomplishing a success rate of 50% to 80% [3]. Despite a reported 11% likelihood of relapse within 2 weeks after treatment [4], GO cases that may be at the risk of decreased vision may be unresponsive to surgical orbital decompression or high-dose corticosteroid therapy (up to 8 g cumulative doses). Such treatments can occasionally result in severe adverse effects, including acute liver failure. As a result, managing corticosteroid-resistant or relapsed cases, influencing 20% to 30% of patients, remains a significant challenge for GO specialists [5].

Self-reactive B cells recognize an autoantigen, specifically the thyroid-stimulating hormone (TSH) receptor found in the orbit and on thyroid epithelial cells, and subsequently secrete cytokines, such as interleukin-6 (IL-6). The release of these pro-inflammatory cytokines leads to the development of peri-orbital edema and exophthalmos. The efficacy of tocilizumab (TCZ) in treating GO is attributed to its ability to specifically inhibit the signaling process mediated by both soluble and membrane-bound IL-6 receptors [6].

Furthermore, patients treated with TCZ exhibited improvements in severity and exophthalmos as assessed by the European Group on Graves' Orbitopathy (EUGOGO) classification. The present study aimed to assess the therapeutic efficacy of TCZ in patients who did not respond to first-line corticosteroid treatments, and to determine the potential of TCZ in providing benefits to patients with milder, less active GO.

## Methods

### Patients, Enrollment Criteria, and Study Design

**Figure 1.**
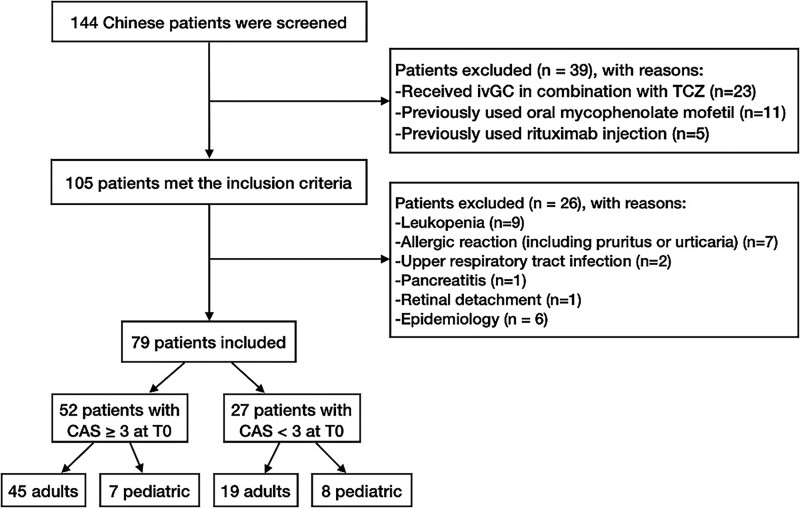
Flow chart of the enrollment and exclusion process for patients.

A retrospective longitudinal study was conducted at a single center between November 2021 and February 2024, where medical records were reviewed. Each patient served as his/her own control. A total of 144 Chinese patients diagnosed with steroid-resistant GO (without DON) or juveniles whose guardians refused steroid treatment were examined and treated with TCZ. Overall, 79 patients met the inclusion criteria, including 15 pediatric patients aged ≤ 18 years. Among them, 52 patients had moderate to severe and active GO. Prior to initiating TCZ treatment, all patients had undergone a minimum of 3 months since their last steroid treatment, to minimize the potential influence of prior medication on the study outcomes. The study excluded patients who stopped treatment due to the following reasons: leukopenia (n = 9), allergic reaction (including pruritus or urticaria) (n = 7), upper respiratory tract infection (n = 2), pancreatitis (n = 1), and retinal detachment (n = 1). Side effects appeared mostly after the second dose. No serious side effects associated with tocilizumab were recorded. TCZ administration was discontinued immediately in the patients who developed side effects. In addition, patients who had received intravenous glucocorticoids (ivGC) in combination with TCZ (n = 23) or had previously used oral mycophenolate mofetil (n = 11) or rituximab injection (n = 5) were also excluded from the study. A flow chart of the enrollment and exclusion process for patients is shown in Fig. 1.

All patients presented with binocular disease and all adult patients had resistant or relapsed disease after receiving first-line ivGC treatment (cumulative dose, 5.48 ± 2.32 g) according to the EUGOGO. The severity of GO was assessed according to the EUGOGO. At the completion of the ivGC treatment, resistant definition was a lack of response to corticosteroids in clinical activity score (CAS), and absence of changes or changes smaller than those defined in any of the previously mentioned parameters. Corticosteroid relapse was identified as change in 2 of the following outcome measures in at least 1 eye: worsening of inflammatory eyelid swelling, worsening of CAS [7] of at least 2 points, increase of proptosis > 2 mm, worsening of lid width of at least 2 mm, worsening of diplopia, deterioration of eye muscle motility, and occurrence of DON, which was measured 12 weeks after corticosteroid administration.

All patients received TCZ intravenously at a dose of 8 mg/kg every 28 days for at least 4 cycles and were followed for an additional period of 23.17 ± 21.08 weeks. All patients were evaluated by a single ophthalmologist at T0 (before TCZ treatment), T4 (the period of disease stabilization: approximately 1 month after 4 cycles), and T5 (follow-up assessment after 4 cycles, 23.17 ± 21.08 weeks).

The study protocol was approved by the Clinical Research Ethics Committee of the Xian Fourth Hospital (Xian, China). Informed consent was obtained from all patients before the prescription of TCZ, as TCZ was used as an off-label indication by the National Medical Products Administration of China (NMPA) for the treatment of GO.

### Data Collection

All the relevant medical data, including demographic data, history of thyroid disease, date of GD and GO diagnosis, whether GD had been treated, smoking status, surgical decompression, TCZ times, and follow-up time were collected. All patients underwent a complete blood test, involving an assessment of thyroid function and autoimmunity (including thyrotropin receptor antibodies [TRAbs], TSH, triiodothyronine, thyroxine, anti-thyroglobulin antibodies, anti-thyroid peroxidase antibodies, thyroglobulin, and IL-6), lipid status, liver function, and a thorough serology examination to exclude the presence of active infectious diseases. All patients had normal thyroid hormone levels at the time of inclusion, and they were monitored during the treatment and follow-up period. Blood measurements were repeated every 4 weeks.

### Outcomes and Assessments

The primary efficacy variables included visual acuity (VA), best corrected visual acuity (BCVA), intraocular pressure (IOP), Hertel value, CAS, and TRAb levels. These variables were measured from baseline throughout the treatment period with TCZ. Other outcome variables were the presence of spontaneous orbital pain, gaze evoked orbital pain, eyelid swelling and erythema, conjunctival redness and chemosis, caruncular swelling, exophthalmos, motility, and diplopia.

Activity and severity before and during TCZ were evaluated by the EUGOGO recommendations using the CAS score. It is the sum of various items and ranges from 0 (no activity) to 10 (maximal activity measured in progression) [8]. CAS is based on the evaluation of 10 items: the presence of spontaneous orbital pain, gaze evoked orbital pain, eyelid swelling and erythema, conjunctival redness and chemosis, caruncular swelling, increase of proptosis > 2 mm during a period of 1 to 3 months, decrease of eye movement in any direction ≥ 8° during a period of 1 to 3 months, and reduction of visual acuity > 1 line on the Snellen chart. For each of the 10 items exposed, 1 point was given. CAS ≥ 3 points indicated active GO at T0, and CAS ≥ 4 points indicated active GO at T4 and T5. The clinical response to TCZ was defined as the improvement in the most affected eye, a reduction of at least 2 measures of a composite index without simultaneous deterioration of any of the ophthalmic parameters of the index. The composite index measures were improvement of eyelid swelling in accordance with the EUGOGO color atlas, a decrease ≥ 2 points in the CAS, improvement in proptosis of at least 2 mm, improvement in lid width of at least 2 mm, improvement in diplopia, and improvement of eye muscle motility > 8°. Low disease activity was defined when the CAS was ≤ 3 points [9].

The Snellen chart was utilized to estimate VA and BCVA. According to this test, normal vision was defined as 20/20 vision. In this study, 20/20 vision (normal vision) was represented as 1.0, while 0/20 vision was expressed as 0.0 [10]. Proptosis was measured in millimeters using the same Hertel instrument. Subjective diplopia was evaluated in the primary and extreme gaze positions.

### Statistical Analysis

Data were analyzed using the GraphPad Prism10.1.2 (GraphPad Inc, USA). Continuous variables with normal distribution are presented as mean ± SD. Normal distribution of continuous variables was assessed with Kolmogorov-Smirnov test. The difference between pre- and post-TCZ administration results were calculated by the paired *t* test or Wilcoxon signed rank test, depending on whether the data with a normal distribution. A value of *P* < .05 was considered statistically significant.

## Results

### Main Clinical Features at First TCZ Administration

In total, 79 Chinese patients, with active bilateral GO who had persistence of disease despite treatment with corticosteroids or who were unable to tolerate systemic steroids, were identified as candidates for TCZ therapy. Table 1 summarizes patient demographics and clinical characteristics.

**Table 1.** Demographic and clinical characteristics of patients with GO treated with Tocilizumab

Characteristic	All patients (n = 79)	Pediatric (≤18 years) (n = 15)	Adults (>18 years) (n = 64)	*P* ^†^
Age, years, mean ± SD	39.87 ± 18.50	12.13 ± 3.58	46.38 ± 13.95	
Sex (%)				
Male	34.2	26.7	36	
Female	65.8	73.3	64.1	
Weight, kg	60.44 ± 15.38	47.43 ± 21.83	63.49 ± 11.72	.0002*
Duration of GD, months, mean ± SD	23.32 ± 27.29	17.70 ± 21.11	24.64 ± 28.53	.38
Duration of TAO, months, mean ± SD	18.42 ± 25.18	14.13 ± 13.02	19.42 ± 27.23	.47
GD has been treated (%)	81	73.3	82.8	
Hypertension (%)	16.5	0	20.3	
Diabetes mellitus (%)	6.3	0	7.8	
Smoking status (%)	3.8	0	4.7	
ivGC (%)	81	6.7	100	
ivGC + Secondary (%)	3.8	0	4.7	
Total IV steroids dose (g)	5.37 ± 2.38	1.5	5.48 ± 2.32	
Surgical decompression (%)	17.7	0	21.9	
TCZ times, mean ± SD	4.87 ± 1.17	5.07 ± 0.88	4.83 ± 1.23	.48
Follow-up, weeks, mean ± SD	23.17 ± 21.08	21.46 ± 25.15	23.61 ± 20.22	.77
TSH levels (uIU/mL)	2.04 ± 2.44	1.32 ± 1.64	2.16 ± 2.54	.57
IL-6 (pg/mL)	14.21 ± 9.78	11.72 ± 7.59	14.58 ± 10.06	.89
TRAb (IU/L)	7.40 ± 9.67	8.65 ± 10.98	7.19 ± 9.51	.6
T3 levels (nmol/L)	1.65 ± .51	1.85 ± 0.79	1.62 ± 0.45	.02*
fT4 levels (pmol/L)	12.38 ± 3.64	12.87 ± 6.50	12.30 ± 3.00	.22
anti-TPOAb (IU/mL)	123.02 ± 252.76	221.34 ± 325.75	106.63 ± 237.62	.04*
TgAbs (IU/mL)	73.44 ± 208.42	231.51 ± 358.52	46.96 ± 160.63	.0003*
TG (ng/mL)	57.80 ± 81.72	65.55 ± 134.30	56.47 ± 70.49	.94

Abbreviations: anti-TPOAb, anti-thyroid peroxidase antibodies; fT4, thyroxine; GD, hyperthyroidism; GO, Graves' orbitopathy; IL-6, interleukin-6; ivGC, intravenous glucocorticoids; T3, triiodothyronine; TAO, thyroid-associated ophthalmopathy; TG, thyroglobulin; TgAbs, anti-thyroglobulin antibodies; TRAbs, thyrotropin receptor antibodies; TSH, thyroid-stimulating hormone.

*Statistically significant. *P*^†^: Pediatric vs Adults.

Among the included patients, 65.8% were female and the overall mean age (± SD) at TCZ initiation was 39.87 ± 18.50 years. Overall, 81% of cases with GD (hyperthyroidism) were treated. One patient had thyroid surgery 1 year prior. Further, 6.3% of patients were diabetics, 16.5% of patients had hypertension, and 3.8% were smokers. In addition, 17.7% of patients underwent surgical decompression 6 months before the TCZ initiation. No patients underwent further medical treatment, including systemic corticosteroids, or underwent surgical intervention for GO while being treated with TCZ. Average time between the onset of GD or GO and the first TCZ administration was 23.32 ± 27.29 and 18.42 ± 5.18 months, respectively. The average number of TCZ administrations was 4.87 ± 1.17, and the average follow-up time after 4 cycles of TCZ treatment was 23.17 ± 21.08 weeks.

### Therapeutic Efficacy of TCZ

The pre-infusion CAS, TRAb levels, and ocular parameters, including VA, BCVA, IOP, and proptosis were evaluated for each patient. These measurements were compared with their respective scores following the initial infusion and after completing 4 cycles of TCZ treatment (Table 2). Table 3 displays changes of the percentage in each component of the CAS and diplopia from T0 to T5.

**Table 2.** Outcomes of GO patients after tocilizumab therapies (mean ± SD)

	All patients										Patients with CAS ≥ 3 T0									
	T0 vs T4 (158 eyes)					T0 vs T5 (104 eyes)					T0 vs T4 (104 eyes)					T0 vs T5 (66 eyes)				
Outcomes	T0	T4	△_1_	*P^†^*	95% CI	T0	T5	△_2_	*P^‡^*	95% CI	T0	T4	△_1_	*P^†^*	95% CI	T0	T5	△_2_	*P^‡^*	95% CI
All Patients (n = 79, 52 patients with CAS ≥ 3)																				
VA	0.51 ± 0.32	0.54 ± 0.34	0.03 ± 0.16	.03*	0.003∼0.05	0.47 ± 0.31	0.54 ± 0.34	0.07 ± 0.22	.003*	0.02∼0.11	0.50 ± 0.30	0.54 ± 0.32	0.05 ± 0.15	.002*	0.02∼0.08	0.44 ± 0.30	0.53 ± 0.33	0.08 ± 0.17	.0001*	0.04∼0.12
BCVA	0.88 ± 0.20	0.90 ± 0.20	0.02 ± 0.15	.62	−0.03∼0.04	0.86 ± 0.21	0.90 ± 0.20	0.03 ± 0.15	.24	−0.02∼0.08	0.87 ± 0.19	0.91 ± 0.15	0.04 ± 0.15	.76	−0.04∼0.05	0.85 ± 0.20	0.89 ± 0.19	0.02 ± 0.14	.55	−0.04∼0.08
IOP, mmHg	18.17 ± 6.20	16.81 ± 4.69	−1.33 ± 6.02	.007*	−2.27∼−0.38	18.42 ± 5.59	17.40 ± 4.81	−1.06 ± 5.89	.07	−2.21∼0.10	18.31 ± 6.43	17.10 ± 4.68	−1.21 ± 6.01	.04*	−2.38∼−0.04	18.98 ± 5.90	18.04 ± 5.34	−0.94 ± 6.22	.23	−2.47∼0.59
Proptosis, mm	18.06 ± 2.56	17.18 ± 2.45	−0.88 ± 1.56	<.0001*	−1.13∼−0.63	18.17 ± 2.52	17.40 ± 2.50	−0.77 ± 2.04	.0002*	−1.17∼−0.37	18.19 ± 2.69	17.21 ± 2.63	−0.98 ± 1.76	<.0001*	−1.32∼−0.64	18.59 ± 2.56	17.55 ± 2.58	−1.05 ± 2.04	<.0001*	−1.55∼−0.54
CAS	2.31 ± 1.64	1.37 ± 1.43	−0.95 ± 1.60	<.0001*	−1.31∼−0.59	2.10 ± 1.61	1.33 ± 1.49	−0.77 ± 1.77	.003*	−1.26∼−0.28	3.35 ± 0.84	1.75 ± 1.53	−1.60 ± 1.50	<.0001*	−2.01∼−1.18	3.21 ± 0.70	1.55 ± 1.58	−1.67 ± 1.34	<.0001*	−2.14∼−1.19
TRAb, IU/L	7.40 ± 9.67	3.36 ± 5.92	−3.97 ± 5.92	<.0001*	−5.47∼−2.57	6.82 ± 8.21	3.60 ± 6.55	−3.76 ± 8.14	.003*	−6.15∼−1.37	9.43 ± 11.08	4.02 ± 7.02	−4.94 ± 6.51	<.0001*	−6.81∼−3.07	8.43 ± 9.45	3.58 ± 6.87	−4.62 ± 10.05	.018*	−8.37∼−0.87
Pediatric (≤18 years) (n = 15, 7 children with CAS ≥ 3)																				
VA	0.54 ± 0.33	0.52 ± 0.33	−0.02 ± 0.17	.52	−0.08∼0.04	0.52 ± 0.32	0.54 ± 0.37	0.02 ± 0.14	.58	−0.05∼0.08	0.39 ± 0.22	0.38 ± 0.27	−0.02 ± 0.17	.67	−0.11∼0.08	0.44 ± 0.29	0.47 ± 0.36	0.04 ± 0.14	.41	−0.06∼0.13
BCVA	0.92 ± 0.22	0.90 ± 0.20	−0.02 ± 0.10	.49	−0.03∼0.06	0.88 ± 0.27	0.90 ± 0.20	0.02 ± 0.12	.53	−0.05∼0.09	0.96 ± 0.09	0.98 ± 0.06	0.02 ± 0.11	.59	−0.06∼0.10	0.94 ± 0.10	0.98 ± 0.07	0.03 ± 0.14	.6	−0.10∼0.16
IOP, mmHg	16.87 ± 4.13	16.86 ± 4.50	−0.09 ± 5.74	.94	−2.27∼2.10	17.79 ± 4.49	19.00 ± 5.70	1.12 ± 7.76	.51	−2.41∼4.66	17.19 ± 4.76	16.94 ± 4.28	−0.24 ± 5.90	.88	−3.65∼3.17	19.57 ± 4.93	20.73 ± 6.80	1.16 ± 9.98	.7	−5.18∼7.50
Proptosis, mm	18.07 ± 2.02	17.60 ± 1.73	−0.47 ± 1.11	.03*	−0.88∼−0.05	18.05 ± 2.01	17.46 ± 1.79	−0.60 ± 1.10	.02*	−1.08∼−0.10	17.86 ± 1.96	17.50 ± 2.03	−0.36 ± 1.15	.27	−1.02∼0.31	18.17 ± 1.90	17.75 ± 2.01	−0.42 ± 1.00	.18	−1.05∼0.22
CAS	1.40 ± 1.55	0.93 ± 1.16	−0.47 ± 1.19	.15	−1.12∼0.19	1.36 ± 1.57	1.18 ± 1.72	−0.18 ± 1.54	.7	−1.22∼0.85	3 ± 0	1.71 ± 1.25	−1.29 ± 1.25	.04*	−2.45∼−0.13	3 ± 0	2.2 ± 2.17	−0.80 ± 2.17	.46	−3.49∼1.89
TRAb, IU/L	8.57 ± 11.05	3.95 ± 5.90	−4.63 ± 6.09	.01*	−8.00∼−1.25	8.65 ± 10.98	2.72 ± 2.88	−5.14 ± 8.94	.12	−12.01∼1.73	13.13 ± 14.74	6.29 ± 8.19	−6.84 ± 7.78	.059	−14.04∼0.36	11.30 ± 17.32	3.87 ± 4.05	−7.43 ± 13.32	.35	−28.62∼13.77
Adults (>18 years) (n = 64, 45 patients with CAS ≥ 3)																				
VA	0.51 ± 0.32	0.55 ± 0.34	0.04 ± 0.15	.005*	0.01∼0.07	0.46 ± 0.31	0.54 ± 0.34	0.08 ± 0.24	.003*	0.03∼0.13	0.51 ± 0.31	0.57 ± 0.33	0.06 ± 0.14	.0003*	0.03∼0.09	0.45 ± 0.30	0.54 ± 0.32	0.09 ± 0.17	.0002*	0.05∼0.14
BCVA	0.87 ± 0.18	0.91 ± 0.15	0.04 ± 0.16	.8	−0.04∼0.05	0.85 ± 0.20	0.90 ± 0.18	0.04 ± 0.17	.34	−0.04∼0.11	0.84 ± 0.20	0.89 ± 0.16	0.05 ± 0.16	.91	−0.05∼0.06	0.83 ± 0.21	0.87 ± 0.21	0.01 ± 0.15	.73	−0.07∼0.09
IOP, mmHg	18.47 ± 6.55	16.86 ± 4.75	−1.61 ± 5.76	.002*	−2.61∼−0.60	18.58 ± 5.85	17.00 ± 4.50	−1.62 ± 5.22	.006*	−2.76∼−0.47	18.48 ± 6.66	17.12 ± 4.76	−1.36 ± 6.04	.04*	−2.62∼−0.09	18.85 ± 6.13	17.40 ± 4.80	−1.40 ± 5.06	.047*	−2.79∼−0.02
Proptosis, mm	18.06 ± 2.68	17.08 ± 2.58	−0.98 ± 1.64	<.0001*	−1.26∼−0.69	18.21 ± 2.65	17.39 ± 2.67	−0.82 ± 2.23	.001*	−1.31∼−0.33	18.24 ± 2.79	17.17 ± 2.71	−1.08 ± 1.83	<.0001*	−1.46∼−0.70	18.69 ± 2.69	17.50 ± 2.70	−1.19 ± 2.19	.0002*	−1.78∼−0.59
CAS	2.53 ± 1.59	1.47 ± 1.47	−1.06 ± 1.67	<.0001*	−1.48∼−0.65	2.29 ± 1.59	1.37 ± 1.44	−0.93 ± 1.81	.002*	−1.50∼−0.36	3.40 ± 0.89	1.76 ± 1.58	−1.64 ± 1.54	<.0001*	−2.11∼−1.18	3.26 ± 0.75	1.43 ± 1.48	−1.82 ± 1.12	<.0001*	−2.26∼−1.39
TRAb, IU/L	7.12 ± 9.39	3.20 ± 5.97	−3.80 ± 5.92	<.0001*	−5.38∼−2.21	6.30 ± 7.35	3.80 ± 7.14	−3.44 ± 8.04	.01*	−6.08∼−0.79	8.85 ± 10.49	3.66 ± 6.85	−4.63 ± 6.33	<.0001*	−6.60∼−2.66	7.65 ± 8.14	3.54 ± 7.25	−5.66 ± 7.07	.0004*	−8.52∼−2.81

Abbreviations: BCVA, best corrected visual acuity; GO, Graves orbitopathy; IOP, intraocular pressure; TRAbs, thyrotropin receptor antibodies; VA, visual acuity.

*Statistically significant; T0 = before TCZ treatment; T4 = after 4 cycles; T5 = follow-up after 4 cycles; *P*† = T0 vs T4; *P*^‡^ = T0 vs T5. △_1_ = T4-T0; △_2_ = T5-T0.

**Table 3.** Percentage of GO patients in the components of the clinical activity score and diplopia treated with tocilizumab

Components of CAS	Patients with CAS at T0 ≥ 3 (n = 52)									All patients (n = 79)								
	All patients (n = 52)			Pediatric (n = 7)			Adults (n = 45)			All patients (n = 79)			Pediatric (n = 15)			Adults (n = 64)		
	T0	T4	T5 (n = 33)	T0	T4	T5	T0	T4	T5 (n = 31)	T0	T4	T5 (n = 54)	T0	T4	T5 (n = 11)	T0	T4	T5 (n = 43)
Spontaneous orbital pain (%)	50.0	9.6	12.1	100.0	14.3	14.3	42.2	8.9	9.7	35.4	6.3	9.3	46.7	6.7	9.1	32.8	6.3	9.3
Gaze evoked orbital pain (%)	61.5	7.7	9.1	57.1	0.0	14.3	62.2	8.9	6.5	41.8	5.1	5.6	26.7	0.0	9.1	45.3	6.3	4.7
Eyelid swelling (%)	92.3	69.2	60.6	85.7	57.1	42.9	93.3	71.1	54.8	67.1	53.2	46.3	40.0	26.7	36.4	73.4	59.4	48.8
Eyelid erythema (%)	50.0	19.2	24.2	14.3	14.3	28.6	55.6	22.2	19.4	32.9	13.9	18.5	6.7	6.7	18.2	39.1	15.6	18.6
Conjunctival redness (%)	50.0	19.2	18.2	28.6	28.6	28.6	53.3	17.8	12.9	34.2	12.7	11.1	13.3	13.3	18.2	39.1	12.5	9.3
Chemosis (%)	21.2	9.6	6.1	14.3	14.3	14.3	22.2	8.9	3.2	13.9	6.3	3.7	6.7	6.7	9.1	15.6	6.3	2.3
Caruncular swelling (%)	9.6	0.0	3.0	0.0	0.0	0	11.1	0.0	3.2	6.3	0.0	1.9	0.0	0.0	0.0	7.8	0.0	2.3
Exophthalmos (%)	—	7.7	9.1	—	14.3	0	—	6.7	9.7	—	5.1	11.1	—	6.7	0.0	—	4.7	14.0
Motility (%)	—	9.6	9.1	—	0.0	0	—	11.1	9.7	—	11.4	9.3	—	0.0	0.0	—	14.1	11.6
VA↓ (%)	—	23.1	30.3	—	28.6	14.3	—	22.2	29.0	—	22.8	27.8	—	26.7	18.2	—	21.9	30.2
Active GO (%)*	100	13.5	14.3	100	14.3	28.6	100	13.3	10.7	65.8	8.9	10.7	46.7	6.7	13.3	70.3	9.4	9.8
Diplopia (%)	53.9	44.2	48.5	0.0	0.0	0.0	62.2	51.1	51.6	53.2	43.0	38.9	6.7	0.0	0.0	64.1	53.1	48.8

Exophthalmos: increase of >2 mm in proptosis; Motility: decrease in uniocular excursion in any one direction of >8 degrees; VA↓: Decreased acuity equivalent to 1 Snellen line.

Abbreviations: GO, Graves orbitopathy; T0, before TCZ treatment; T4, after 4 cycles; T5, follow-up after 4 cycles.

^*^Percentage of active GO: A CAS ≥ 3 points indicated active GO at T0, and CAS ≥ 4 points indicated active GO at T4 and T5.

**Figure 2.**
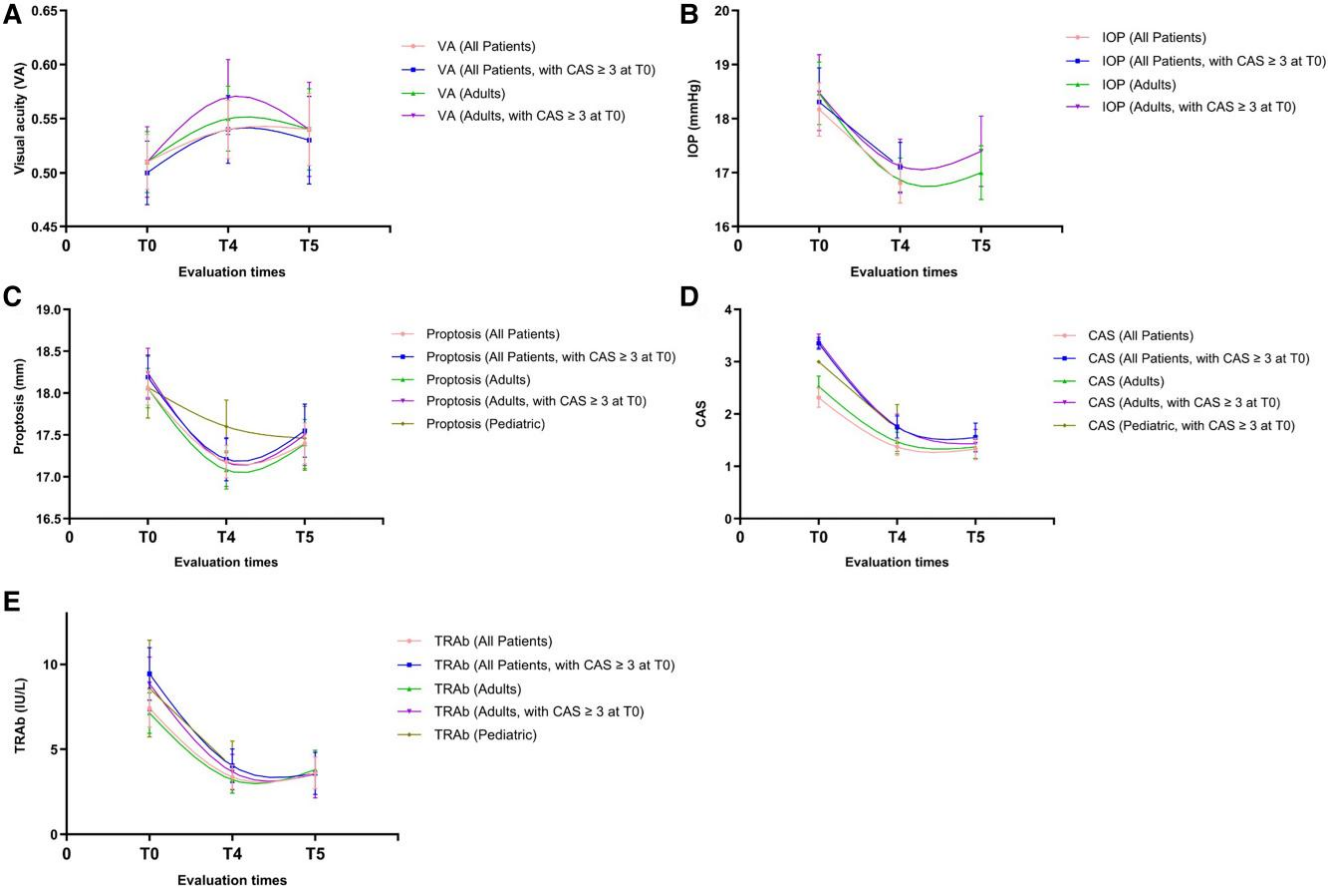
Improvement of the main ocular parameters as well as clinical activity score (CAS) and thyrotropin receptor antibody (TRAb) after tocilizumab therapy (*P* < .05 compared with baseline, Table 2). (A) Visual acuity (VA) evolution; (B) Intraocular pressure (IOP) evolution; (C) Proptosis evolution; (D) CAS evolution; (E) TRAb evolution. Data were expressed as mean ± SE.

Table 2 compares critical parameters and scores before and after treatment with TCZ, including T0 vs T4 and T0 vs T5. Except for the IOP of all patients in T0 vs T5, the VA, IOP, Hertel value, CAS, and TRAb in all patients and in the adult group (> 18 years of age) showed clinically significant rapid and lasting improvement in T0 vs T4 and T0 vs T5 (*P* < .05). In the pediatric group (≤ 18 years), the Hertel value and TRAb in T0 vs T4, the Hertel value in T0 vs T5, and the CAS (active GO at T0) in T0 vs T4 exhibited clinically significant improvement (*P* < .05). The improvement of BCVA after treatment in all groups was not statistically significant (Fig. 2).

In the adult group, 4 cycles TCZ treatment significantly improved the VA by 0.04 ± 0.15 and 0.06 ± 0.14 (active GO at T0), reduced the IOP by 1.61 ± 5.76 and 1.36 ± 6.04 mmHg (active GO at T0), decreased the Hertel value by 0.98 ± 1.64 and 1.08 ± 1.83 mm (active GO at T0), reduced the CAS by 1.06 ± 1.67 and 1.64 ± 1.54 points (active GO at T0), attenuated the TRAb levels by 3.80 ± 5.92 and 4.63 ± 6.33 IU/L (active GO at T0) (*P* < .05). At the end of follow-up, the VA was significantly improved by 0.08 ± 0.24 and 0.09 ± 0.17 (active GO at T0), the IOP was reduced by 1.62 ± 5.22 and 1.40 ± 5.06 mmHg (active GO at T0), the Hertel value was diminished by 0.82 ± 2.23 and 1.19 ± 2.19 mm (active GO at T0), the CAS was reduced by 0.93 ± 1.81 and 1.82 ± 1.12 points (active GO at T0), TRAb levels were reduced by 3.44 ± 8.04 and 5.66 ± 7.07 IU/L (active GO at T0) (*P* < .05). The diplopia (%) decreased from 64.1% to 48.8% and 62.2% to 51.6% (active GO at T0). The decrease in uniocular excursion in any one direction of ≥ 8° (0.0% vs 9.7%) was increased (active GO at T0). Additionally, 60% and 71.4% of the adult group (active GO at T0) experienced a decrease in CAS ≥ 2 points at T4 and T5, respectively. Regarding low activity disease (CAS < 4 points), 86.7% and 89.3% of the adult group (active GO at T0) reached the response criterion at T4 and T5, respectively.

In the pediatric group, 4 cycles of TCZ treatment significantly reduced the Hertel value by 0.47 ± 1.11 mm, TRAb levels by 4.63 ± 6.09 IU/L, and the CAS by 1.29 ± 1.25 points (active GO at T0) (*P* < .05). At the end of follow-up, the Hertel value was reduced by 0.60 ± 1.10 mm (*P* = .02). The diplopia (%) decreased from 6.7% to 0%. Moreover, 42.9% and 60% of the pediatric group (active GO at T0) experienced a decrease in CAS ≥ 2 points at T4 and T5, respectively. Regarding low activity disease (CAS < 4 points), 85.7% and 60% of the pediatric group (active GO at T0) reached the response criterion at T4 and T5, respectively.

## Discussion

Within the realm of inflammatory orbitopathies, GO represents a significant complication of GD, resulting in high levels of morbidity and, in some cases, vision loss for patients. To date, only some observational studies have demonstrated the beneficial effects of TCZ on patients with GO who were resistant to corticosteroids. These studies were inherently limited, and their small sample size is noteworthy, since they mainly included no more than 18 patients [9, 11-14]. Only 3 studies had a sample size exceeding 18, while fewer than 54 cases were involved [15-17]. The cohort in Pérez-Moreiras' study [15] was heterogeneous, as patients were included regardless of their current treatment status. In contrast, in the present study, patients had undergone alternative treatments for at least 3 months before the initiation of TCZ and did not experience any clinical improvement. This indicates that the outcomes observed were not influenced by prior intravenous steroid treatment, as such treatment typically yields a peak benefit duration of 6 to 12 weeks in patients with moderate to severe active GO [18]. In the study by Sánchez-Bilbao [16], some patients received conventional immunosuppressive drugs combined with TCZ, and therefore, the net treatment effect remained elusive. In the present study, patients who had received conventional immunosuppressive drugs in combination with TCZ or had prior TCZ treatment were also excluded. Additionally, the dosage of TCZ was not standardized across all patients [17]. In the present study, all patients received TCZ intravenously as the standardized treatment.

This study encompassed the largest series of patients with GO undergoing TCZ in real-world conditions, corroborating the results obtained in the only randomized controlled trial (RCT) [15]. Both studies suggest a significant improvement of inflammation and proptosis in patients with moderate or severe active GO. Besides, the results of this study indicated that TCZ could be utilized as an effective and safe treatment for Chinese patients, involving those adults with active moderate to severe steroid-resistant GO diseases, as well as GO patients including pediatrics. Most patients displayed a rapid improvement in the main ocular parameters as well as CAS and TRAb after TCZ therapies (Table 2, Fig. 2).

The main finding was a significant reduction in CAS outcomes during treatment with TCZ. The reduction in CAS scores was accompanied by the decline in TRAb levels, which remained higher than normal (< 1.75 IU/L) at T5, despite them being clinically inactive. Based on the curve's trajectory of Fig. 2D and 2E, it was revealed that the clinically inactive state was stabilized after T5. Prior research [19] demonstrated that the TRAb measurement could serve as an exceptional tool for evaluating the prognosis and assessing the response of active GO to treatment [20]. In order to measure the reduction in clinical activity after starting TCZ and throughout the follow-up, CAS was combined with other disease activity parameters, such as BCVA, VA, IOP, and the Hertel value. As this study excluded patients with DON, the improvement of BCVA in all groups after treatment was not statistically significant. The present study disclosed the improvement of VA, IOP, and the Hertel value in the adult group, regardless of whether there was active GO at T0. Although they rebounded during the follow-up period, they were still significantly improved at the end of the follow-up (Fig. 2A-2C). The rebound of VA and IOP was different from the linear results of Bilbao [16], while the rebound of the Hertel value was different from the linear results of Boutzios [13]. The rebound of these parameters may be attributed to the fact that TCZ modifies the course of GO by inhibiting both the soluble and membrane forms of the IL-6 receptor. However, the inhibitory effect is not sustained after drug withdrawal. Nevertheless, the joint evaluation of parameters, such as CAS, TRAb, VA, IOP, and the Hertel value demonstrates an overall improvement, confirming the efficacy of tocilizumab. The lasting effect of tocilizumab could improve CAS [15].

In the present study, the adult group (active GO at T0) experienced an improvement in CAS of 1.82 ± 1.12 points at T5. Moreover, 60% and 71.4% of the adult group (active GO at T0) experienced a reduction in CAS ≥ 2 points at T4 and T5, respectively. These results are in line with those reported by Sánchez-Bilbao [16], in which 75.3% of the affected eyes had a decrease in CAS ≥ 2 points at 12 weeks. The clinical and biochemical improvements observed after treatment with TCZ were more significant than those observed in the commonly described natural history of GO [21] and in the placebo groups of various studies [15, 22, 23]. For instance, in the RCT [15] conducted by Pérez-Moreiras, 58.8% of patients who received placebo experienced an improvement in CAS > 2 points at 16 weeks. In the present study, 86.7% of the adult group (active GO at T0) met the response criteria (low activity disease, CAS < 4 points) at T4, which is significantly higher than the 35.2% observed in the placebo group of the RCT [15]. Furthermore, in the present study, 89.3% of the affected eyes exhibited low disease activity at T5, compared with 47.1% of patients in the placebo group during the RCT [15] at week 40. This finding reinforces the notion that the clinical improvement is attributable to TCZ treatment and should not be misconstrued as spontaneous improvement (Table 2).

GO is found in approximately one-third of patients with juvenile Graves hyperthyroidism [24]. The incidence rate of GO in children is between 1.7 and 3.5 cases per 100 000 individuals per year, with rates varying between 0.79 and 6.5 cases per 100 000 children [24]. GO is more prevalent in girls than in boys, and it primarily affects adolescents aged 11 to 18 years (accounting for 68.2% of cases), compared with children younger than 11 years (31.8%) [24]. At present, glucocorticoid serves as the primary treatment for pediatric GO; however, its efficacy is subject to debate, and it may pose risks of severe adverse reactions [25, 26]. The safety and efficacy of TCZ in pediatric patients have not been assessed [5, 27]. Graefe sign, eyelid retraction, proptosis, and involvement of soft tissue are the predominant symptoms in pediatric patients, while VA and IOP typically remain unaffected [28]. This could explain why TCZ has no significant effect on VA and IOP in the pediatric group (Table 2). During puberty, a higher incidence of serious complications, such as restrictive strabismus and exposure keratopathy, was noted [29]. It was found that after restoring euthyroidism in pediatric patients with GO, position and motility disturbances were mostly improved, while proptosis persisted or was alleviated [30]. In the present study, the pediatric group showed improvement of the Hertel value and the degree of exophthalmos was reduced after T4, which could be stabilized after T5 (Fig. 2C). Regarding the improvement of the CAS (active GO at T0) and the TRAb levels at T4 (Table 2), 60% of the pediatric group (active GO at T0) exhibited a decrease in CAS ≥ 2 and CAS < 4 points (low activity disease) at T5. This confirmed the efficacy of tocilizumab in pediatrics, and the lasting effect of tocilizumab could improve proptosis. However, between phase T0 and T4 exophthalmos (0.0% vs 14.3%) and decreased acuity equivalent to 1 Snellen line (0.0% vs 28.6%) were increased in the pediatric group (active GO at T0) (Table 3), which suggest that TCZ should be used with some caution in the pediatric population.

There are several potential limitations in the present study, in which the most notable limitation is the small number of pediatric patients included. Additionally, the observed clinical improvement in patients could potentially be attributed to both the effect of TCZ treatment and the natural progression of the disease. However, the mean follow-up time, which was 23.17 ± 21.08 weeks, did not allow us to determine the recurrence rate after discontinuation of TCZ treatment. Therefore, further RCTs concentrating on juvenile GO along with a longer follow-up are necessary to assess recurrence rates after discontinuing TCZ and to evaluate any long-term changes in outcomes.

In conclusion, TCZ may be a therapeutic option for adult patients with active, corticosteroid-resistant, moderate to severe GO. It also has the potential to benefit juvenile GO patients, with some caution.

## Data Availability

Some or all datasets generated during and/or analyzed during the current study are not publicly available but are available from the corresponding author on reasonable request.

## Abbreviations

BCVA: best corrected visual acuity
CAS: clinical activity score
DON: dysthyroid optic neuropathy
EUGOGO: the European Group on Graves' Orbitopathy
GD: Graves disease
GO: Graves orbitopathy
ivGC: intravenous glucocorticoids
IL-6: interleukin-6
IOP: intraocular pressure
RCT: randomized controlled trial
T0: before tocilizumab treatment
T4: the period of disease stabilization (approximately 1 month after 4 cycles)
T5: follow-up after 4 cycles
TRAbs: thyrotropin receptor antibodies
TCZ: tocilizumab
TSH: thyroid-stimulating hormone
VA: visual acuity
